# Radial glial cells in the adult dentate gyrus: what are they and where do they come from?

**DOI:** 10.12688/f1000research.12684.1

**Published:** 2018-03-05

**Authors:** Daniel A. Berg, Allison M. Bond, Guo-li Ming, Hongjun Song

**Affiliations:** 1 Department of Neuroscience and Mahoney Institute for Neurosciences, Perelman School for Medicine, University of Pennsylvania, Philadelphia, PA, 19104, USA; 2 Department of Cell and Developmental Biology, Perelman School for Medicine, University of Pennsylvania, Philadelphia, PA, 19104, USA; 3 Institute for Regenerative Medicine, Perelman School for Medicine, University of Pennsylvania, Philadelphia, PA, 19104, USA; 4 The Epigenetics Institute, Perelman School for Medicine, University of Pennsylvania, Philadelphia, PA, 19104, USA

**Keywords:** radial glial cells, dentate gyrus, stem cell heterogeneity

## Abstract

Adult neurogenesis occurs in the dentate gyrus in the mammalian hippocampus. These new neurons arise from neural precursor cells named radial glia-like cells, which are situated in the subgranular zone of the dentate gyrus. Here, we review the emerging topic of precursor heterogeneity in the adult subgranular zone. We also discuss how this heterogeneity may be established during development and focus on the embryonic origin of the dentate gyrus and radial glia-like stem cells. Finally, we discuss recently developed single-cell techniques, which we believe will be critical to comprehensively investigate adult neural stem cell origin and heterogeneity.

## Introduction

The dentate gyrus (DG) is a V-shaped structure in the hippocampus, which is located in the medial temporal cortex of mammals. The addition of newborn neurons to the DG, unlike other areas of the brain, such as the neocortex where neurons are generated only during embryonic development, continues throughout life through a process named adult neurogenesis ^1, 2^. Interestingly, adult neurogenesis in the DG has been observed in all studied mammals, including humans, suggesting that there may be some evolutionarily conserved function of adult hippocampal neurogenesis ^3– 6^. Indeed, animal models have shown that adult neurogenesis in the DG plays important roles in both cognitive and affective behaviors, such as spatial memory learning and retention, pattern separation, and memory clearance ^7– 11^.

Adult-born neurons in the DG are derived from a population of neural stem cells (NSCs) named radial glia-like cells (RGLs) ^1^. RGLs express some astrocyte and stem cell markers and can generate both granule neurons and astrocytes but typically not oligodendrocytes ^12– 15^. These RGLs retain the capacity to divide and generate new neurons throughout life, even in the aging animal ^16, 17^. Recently, more and more studies have shown that rather than being a homogeneous population of identical cells, the RGL population is made up of multiple subpopulations of RGLs that differ in their morphology and how they react to external stimuli ^18^. In this review, we discuss recent discoveries concerning adult neurogenesis in the DG and focus on RGL heterogeneity. Furthermore, we review current knowledge about the embryonic and early postnatal development of the DG and RGL origins given that NSC heterogeneity may be established during development. Finally, we discuss current single-cell analysis techniques that could be used to answer a multitude of remaining questions that concern RGL heterogeneity and its origin.

## Classic homogeneous radial glia-like cell population model

**Figure 1.**
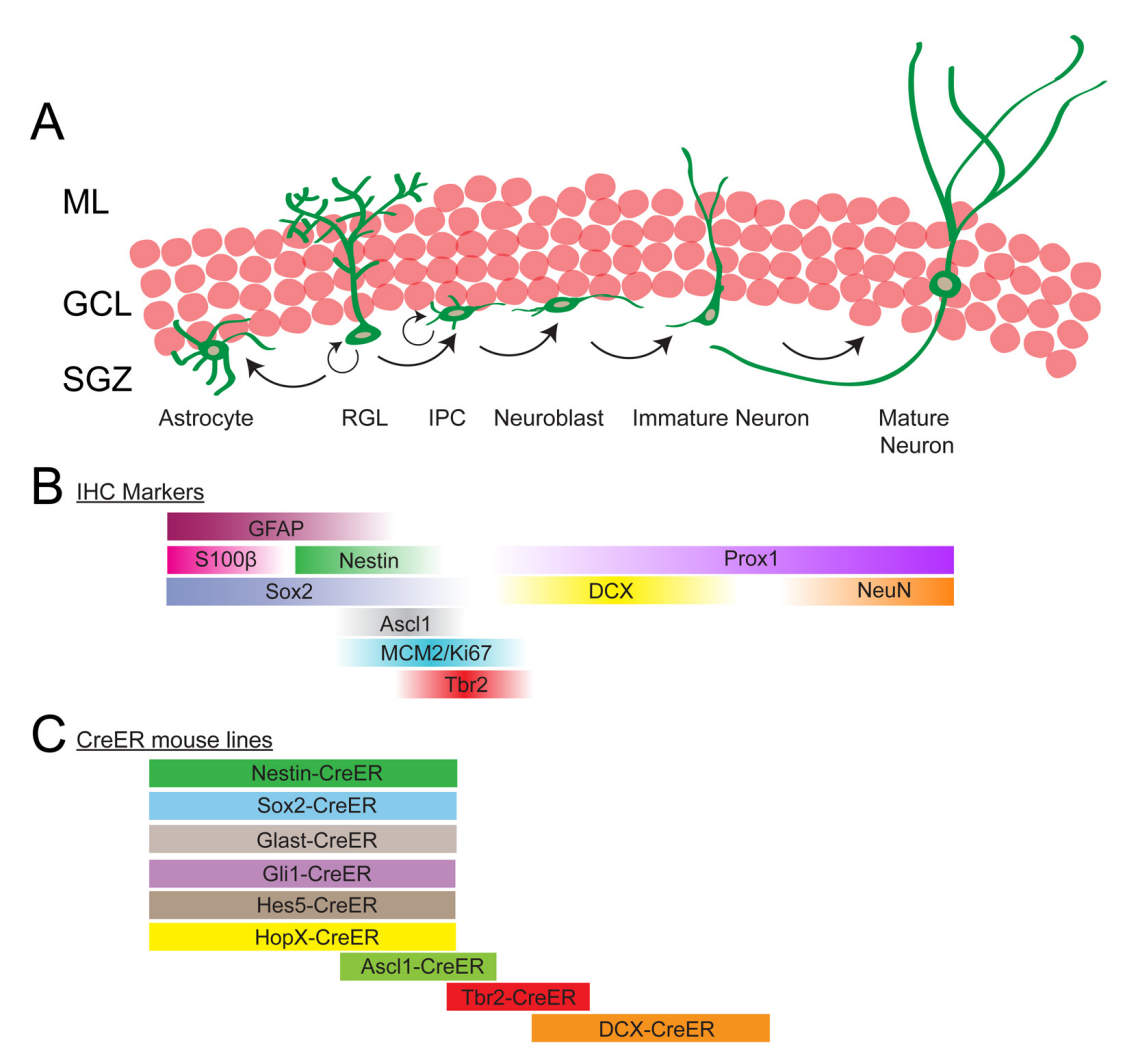
Lineage progression of adult neurogenesis. ( **A**) Radial glia-like cells (RGLs), situated in the subgranular zone (SGZ) of the dentate gyrus (DG), have the potential to both self-renew and give rise to astrocytes and neurons. Each RGL has a bushy process that extends through the granule cell layer (GCL) and ends in the molecular layer (ML). In the process of generating neurons, RGLs divide to generate intermediate progenitor cells (IPCs), which are highly proliferative and lineage-restricted to the neuronal fate. IPCs progress through a series of steps and eventually differentiate into mature neurons which integrate into the existing neuronal networks. ( **B**) Immunohistochemical (IHC) markers that can be used to distinguish different stages of the lineage progression in adult neurogenesis in the DG. ( **C**) Many Cre-ER transgenic mouse lines have been used to label different cell types throughout the process of neurogenesis. Most of the Cre-ER lines that induce recombination in RGLs also label astrocytes but to varying degrees. Tbr2-CreER and doublecortin (DCX)-CreER lines label neural precursors with no contamination of astrocytes or neural stem cells, while the Ascl1-CreER line labels both RGLs and IPCs.

Multiple studies have indicated that RGLs (also known as type 1 cells) are putative NSCs, which generate dentate granule neurons in the adult DG ^14, 15, 19, 20^. RGLs are similar in appearance to radial glial cells of the embryonic brain and share many markers expressed by NSCs in the embryo, including Nestin, glial fibrillary acidic protein (GFAP), and sex-determining region Y-box 2 (Sox2) ^13^. The somas of the RGLs are situated in the subgranular zone (SGZ) of the DG, a region between the granule cell layer (GCL) and the hilus ( Figure 1A). RGLs have a bushy radial process, which extends through the GCL to the molecular layer and terminates with end-feet on both synapses and vasculature ^21, 22^.

RGLs are generally a quiescent population of precursor cells that only occasionally divide ^14, 23, 24^. However, when RGLs undergo cell division, they can divide either symmetrically or asymmetrically multiple times, suggesting that they retain the capacity to self-renew ^13, 20^. During the process of neurogenesis, RGLs divide and give rise to intermediate progenitor cells (IPCs), which express the T-box brain protein 2 (Tbr2/Eomes) ( Figure 1A, B). IPCs have short multipolar processes, are lineage-restricted, and undergo limited rounds of division ^25, 26^. IPCs then give rise to bipolar neuroblasts, which express doublecortin (DCX). Neuroblasts migrate tangentially along the SGZ before migrating short distances radially into the GCL, where they mature into functional Prox1-positive dentate granule neurons ^15^.

Quiescent RGLs are difficult to label using developmental lineage-tracing methods, such as thymidine analogues—5-bromo-2'-deoxyuridine (BrdU) and 5-ethynyl-2'-deoxyuridine (EdU)—or retrovirus, because these techniques label dividing cells only ^27^. However, RGLs can be labeled using multiple tamoxifen-inducible CreER ^T2^ mouse lines ( Figure 1C). Interestingly, the use of different mouse lines has started to reveal the complexity and heterogeneity of the progenitor cells in the adult DG. For example, a study using the Nestin-CreER ^T2^ and glutamate aspartate transporter (Glast)-CreER ^T2^ mouse lines has shown that while the cells labeled using both lines contribute to neurogenesis under homeostasis, only Glast-CreER ^T2^-labeled RGLs contributed to increased proliferation after running and repopulation after injury ^28^. Additionally, studies using the Hes5-CreER ^T2^ and Sox2-CreER ^T2^ lines have suggested the presence of a horizontal and non-radial NSC in the DG ^19, 20^. These and other studies have provided evidence that subpopulations of RGLs with different properties coexist in the adult DG, and current work has been focusing on identifying and distinguishing these populations.

## Modern heterogeneous radial glia-like cell population model

Given the accumulating evidence that RGLs are not identical in the adult mouse DG ^28, 29^, a central question in the field of adult neurogenesis arises: how do discrete subtypes of RGLs in the niche differ in their capacity to self-renew and differentiate? For example, it is possible that a distinct population of RGLs is responsible for generating neurons while another is responsible for generating astrocytes. These possibilities have not been exhaustively explored yet, but recent data clearly suggest that the SGZ consists of RGLs with different morphologies and behaviors.

The adult mouse DG can be divided along its longitudinal axis into the septal pole (the dorsal region) and the temporal pole (the ventral region) ^30^. Interestingly, the septal and temporal DGs function differently, from the systems level all the way down to the molecular level ^31^. For example, the septal region of the DG has been shown to be involved with spatial learning while the temporal region is involved in emotional behavior and motivation ^32, 33^. Similarly, many properties of neurogenesis are dependent on their location along the septo-temporal axis of the DG. For example, the density of RGLs and neuroblasts is lowest in the temporal region of the DG ^34^. Additionally, the tempo of neurogenesis is faster in the septal region of the DG ^35^. At the molecular level, there is a gradient in the expression of the Wingless/INT (Wnt) inhibitor Frizzled-related protein 3 (sFRP3), and the highest expression is observed in the temporal pole ^36^. Deletion of sFRP3 leads to activation of RGLs, suggesting a potential niche mechanism for generating regional heterogeneity in the adult DG ^37^. *In vitro* studies also suggest different neurosphere-forming capacities of the neural progenitors along the septo-temporal axis. Treatment of neurospheres with norepinephrine and KCl was shown to increase the number of neurospheres generated from the temporal region, while neurospheres from the septal region were unaffected ^38^. These data indicate that there are distinct populations of RGLs in the adult DG, which respond differently to niche signaling.

Currently, there are no defined NSC markers to distinguish these different RGL populations *in vivo*. Instead, morphological differences have been used as one approach to distinguish between different subtypes of RGLs. A recent study used careful analysis of confocal images to show that RGLs can be divided into two classes on the basis of their morphology ^29^. Cells of the most common type, termed type α cells in the study, possess longer and less branched processes compared with the less prevalent type β cells. Lineage tracing showed that the type α cells could give rise to neurons, astrocytes, and type β cells but that type β cells did not proliferate. This suggests that type α cells are hierarchically above type β cells, but it is not known whether all type α cells have the potential to give rise to the type β cell over time. Future studies should investigate whether the population of type α cells is homogeneous or consists of multiple cell types.

Return to quiescence after cell division is considered a hallmark of slowly cycling, self-renewing adult stem cells and can be assessed using thymidine analogues, such as BrdU and EdU. These labels get incorporated into the DNA of cells during the S phase of the cell cycle. If the cell continues to undergo multiple divisions, the label gets diluted to an undetectable level. In contrast, the presence of label-retaining RGLs after long chase periods indicates that these cells have returned to quiescence after dividing when the EdU or BrdU was administered. For example, one study administered a single injection of BrdU to Nestin-GFP mice and showed that the RGLs that incorporated BrdU quickly diluted the label, and no label-retaining RGLs were found after a 15-day chase ^16^. This can be put in contrast to other studies, which used longer BrdU pulses and found label-retaining RGLs after longer chases ^12, 19, 39^. Thus, the ability to label RGLs that return to quiescence depends on the experimental paradigm used. Another reason for the conflicting conclusions concerning the existence of label-retaining RGLs is because most studies have been performed at the population level, where rare populations may get overlooked. For example, it is possible that not all RGLs are able to return to quiescence and that those that can return to quiescence represent a small subpopulation of RGLs in the adult DG. Therefore, a high-resolution understanding of the heterogeneity in the SGZ will require a combination of techniques, including single-cell lineage tracing of specific subpopulations.

Clonal analysis using the Nestin-CreER ^T2^ mouse line has revealed that at least some Nestin ^+^ RGLs can self-renew multiple times and are multipotent (generating both neurons and astrocytes) under physiological conditions in the adult DG. Importantly, the Nestin-CreER ^T2^-labeled RGLs are able to return to quiescence after activation ^13^. Nestin protein is present in most, if not all, RGLs in the adult DG, but it is not known whether the cells labeled under clonal analysis conditions represent the majority of the RGL pool or compose a small subpopulation. It should also be noted that different Nestin-CreER ^T2^ lines have varying specificity and could potentially label different subtypes ^40^.

**Figure 2.**
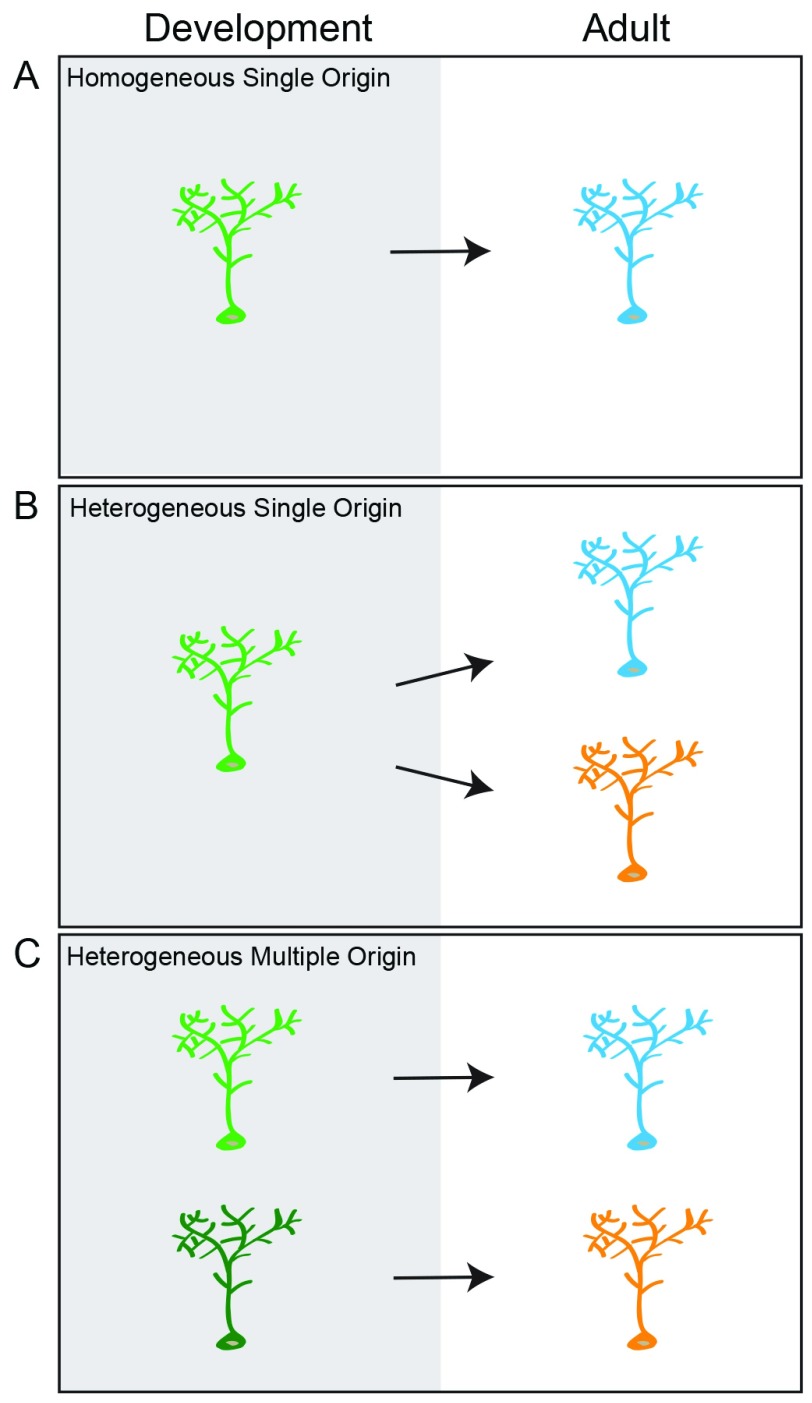
Developmental origin of adult neural stem cell heterogeneity. There are three potential models for the developmental origin of adult neural stem cells. ( **A**) Homogeneous single origin: all radial glia-like cells (RGLs) in the adult dentate gyrus (DG) belong to a single population of stem cells that have similar potentials and make similar fate choices. These cells have a common developmental progenitor. ( **B**) Heterogeneous single origin: there are multiple populations of RGLs in the adult DG that have distinct potentials and may make different fate choices, but these cells have a common developmental precursor. ( **C**) Heterogeneous multiple origin: there are distinct populations of RGLs in the adult DG, and they are generated by different lineage-restricted precursors.

Stem cell heterogeneity has been more rigorously studied in the adult SVZ. The putative NSCs in the adult SVZ are the type B1 cells. These cells express GFAP and have a radial morphology. The type B1 cells can be divided into two groups—the quiescent neural stem cells (qNSCs) and activated neural stem cells (aNSCs)—which can be distinguished by their transcriptional profiles ^41, 42^. Quiescence was associated with a lack of Nestin expression and high glycolytic and lipid metabolism, whereas activation was associated with upregulation of Nestin expression and high protein synthesis and differentiation priming. The qNSCs give rise to the aNSCs, which in turn generate progeny that migrate to the olfactory bulb and become different types of granular cells and periglomerular interneurons ^43, 44^. Different types of interneurons are derived from specific subpopulations of type B1 cells located in distinct areas of the ventricular wall. Type B1 cells from different regions of the ventricular wall remain restricted to their lineages even when transplanted into other areas of the ventricular wall, suggesting that they are intrinsically different from each other ^44, 45^. Furthermore, SVZ progenitors that generate astrocytes are found in distinct domains of the SVZ ^46^. Clonal lineage tracing from development into adulthood has revealed that regionally specified embryonic NSCs give rise to distinct subpopulations of type B1 cells, suggesting that heterogeneity in the adult SVZ is established embryonically ( Figure 2C) ^47^. Embryonic DG development has not been studied as extensively as cortical development, and it will be necessary to examine the ontogenesis of the DG to get a complete understanding of when and how adult RGL heterogeneity is established.

## Origin of adult neural stem cells in the dentate gyrus

**Figure 3.**
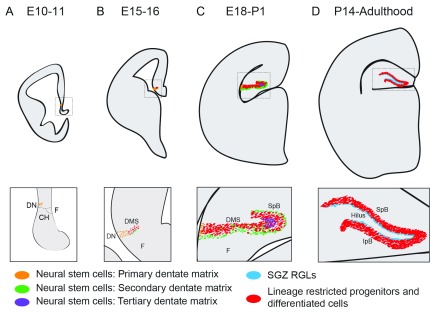
Development of the dentate gyrus. ( **A**) Around embryonic day 10–11 (E10–11), the putative dentate neuroepithelium is situated adjacent to the dentate notch (DN), a small indentation of the ventromedial ventricular wall, which in turn is placed caudal to the cortical hem (CH), neighboring the fimbria (F). These neural stem cells (NSCs) are sometimes referred to as the primary dentate matrix. ( **B**) During embryonic neurogenesis (E15–16), a stream of neural progenitors appears medial to the DN forming the dentate migratory stream (DMS). The DMS consists of both lineage-restricted neural progenitor cells (Tbr2 ^+^) and NSCs (expressing Sox2, Nestin, and GFAP). The DMS leads to the dentate primordium. ( **C**) At about E18, the upper blade of the dentate gyrus (also called the suprapyramidal blade, or SpB) starts to form and contains post-mitotic Prox1 ^+^ neurons, as well as a layer of NSCs that are located subpially, on the upper part of the SpB blade. These NSCs are defined as the secondary dentate matrix, and the NSCs ventral to the SpB are termed the tertiary dentate matrix. ( **D**) At about postnatal day 14 (P14), both the SpB and the infrapyramidal blade (IpB) have formed. At this stage, the secondary and tertiary matrices are gone, and the remaining NSCs—now referred to as radial glia-like cells (RGLs)—are located in the subgranular zone (SGZ), on the border between the granule cell layer and the hilus. This structure and morphology are maintained throughout the rest of the animal’s life.

The origin of the adult RGLs in the DG remains largely unknown. Population studies using thymidine analogues, reporter mice, and immunohistological methods suggest that DG precursors originate from a region called the primary dentate neuroepithelia or primary dentate matrix. Identified by Altman and Bayer ^48^, this putative origin of the DG is situated around the dentate notch, a small indentation in the ventricular wall of the medial pallium, which is visible at embryonic day 11 (E11) in mice ( Figure 3A) ^49, 50^. At around E15, a stream of cells, seemingly originating from this area, start to migrate toward the pial surface into the dentate primordium. This stream, called the dentate migratory stream (DMS), contains both GFAP ^+^Sox2 ^+^ NSCs and Tbr2 ^+^ IPCs ( Figure 3B) ^50, 51^. The proliferating cells in the DMS are termed the secondary dentate matrix ^48^ ( Figure 3A, B). At around E18, Prox1 ^+^ granule neurons appear in what will become the suprapyramidal blade (SpB) of the DG ( Figure 3C) ^49, 52^. At this stage, the secondary dentate matrix is found on the outside of the granule cell layer, while the proliferating NSCs in the hilar region are termed the tertiary dentate matrix. It has been hypothesized that the primary and secondary dentate matrices contribute to embryonic neurogenesis while the tertiary matrix generates the adult RGLs ^52– 54^. It is possible that the secondary matrix generates granule cells in the outer layer of the DG, since work using mouse chimeras has shown that granule cells in this part of the DG are derived from a different pool of progenitors compared with the neurons in the inner layers ^55^.

Although no lineage-tracing studies have examined the early embryonic origin of adult DG RGLs, fate mapping studies using the Gli1-CreER mouse line have shown that a subset of developmental precursors to adult RGLs become sonic hedgehog-responsive around E17.5 ^56^. In this study, Li *et al*. observed that the sonic hedgehog-responsive cells were located in the ventral hippocampus and that these cells migrated into the dorsal hippocampus and generated neurogenic RGLs in the adult animal, suggesting a ventral-to-dorsal NSC migration pattern ^56^.

At postnatal day 14 (P14), the infrapyramidal blade (IpB) has formed, giving the DG its characteristic V-shape ( Figure 3D). By this time, the secondary matrix has disappeared with most of the proliferating NSCs found in the SGZ, where they remain into adulthood ^50, 52^. Immunohistochemical analysis of known markers for adult neurogenesis has suggested that the SGZ is morphologically adult-like by P14, but comprehensive analysis of the potential of individual NSCs during different stages of development is still lacking ^52^.

**Figure 4.**
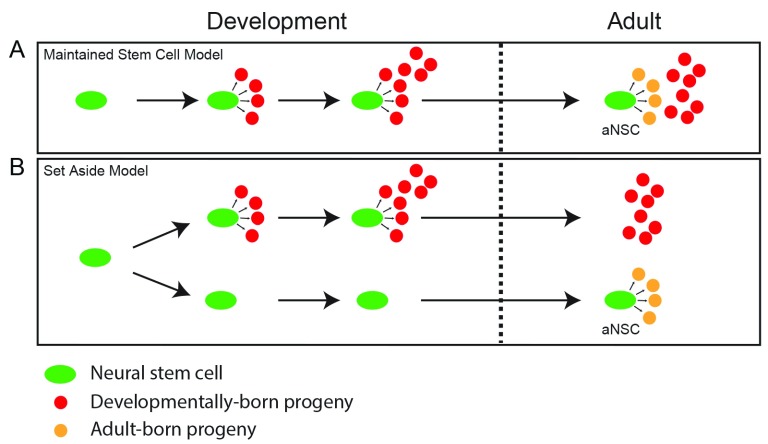
Developmental origin of adult neural stem cells (NSCs). There are two models that explain how adult NSCs are generated during development. It should be noted that these models are not mutually exclusive but can coexist. ( **A**) Maintained stem cell model: NSCs that produce mature cell types to populate the dentate gyrus (DG) during development remain in the adult DG and transition into more quiescent activated NSCs (aNSCs) which generate neurons in the adult DG. ( **B**) Set aside stem cell model: a subset of NSCs are set aside in a quiescent state during development and do not participate in populating the DG with mature cell types. Once the DG is formed, these NSCs are the aNSCs and can reactivate to generate neurons in the adult DG.

In the adult SVZ, type B1 cells are derived from developmental radial glia cells ^57^. In the original model, NSCs that generated neurons throughout development became type B1 cells in the adult and retained their NSC properties ( Figure 4A) ^58, 59^. However, two recent studies have shown that NSCs that ultimately give rise to adult type B1 cells may contribute to developmental neurogenesis to a limited degree but are largely set aside to become quiescent at E13.5–E15.5 until they become active again postnatally ( Figure 4B) ^47, 60^. To examine the early contribution (before E15.5) of the developmental precursors of the adult type B1 cells, Fuentealba *et al*. made use of a retroviral-mediated lineage-tracing method, in which the NSCs were labeled with a genetic barcode ^47^. Sequencing of cells from different areas of the brain revealed that some type B1 cells shared a common progenitor with neurons in other brain areas, including the cortex and striatum. However, as of yet, the identity and location of the developmental type B1 cell precursor are not known, and there is no way to distinguish between these cells and the other more transient developmental NSCs. RNA sequencing of the type B1 cell precursors could reveal novel prospective markers, which then could be used to target these cells for lineage tracing *in vivo*.

Some key aspects of DG development are strikingly different from embryonic cortical development ^61^. First, most neurogenesis in the developing cortex takes place during the embryonic stage, whereas neurogenesis in the DG occurs mostly postnatally, and peak neurogenesis takes place during the first two weeks after birth ^52^. Secondly, the NSCs in the developing cortex and the adult SVZ are continuously in contact with the cerebrospinal fluid (CSF) in the ventricular system, whereas NSCs in the developing and adult DG migrate away from the ventricular niche and must maintain their stem cell properties without contact with the CSF. These traits make the development of the DG exceptional because a neurogenic niche that can support NSCs must be maintained in the postnatal brain, which is less conducive to precursor maintenance, and away from the ventricular system. Understanding how a neurogenic environment is established away from the ventricles in the adult brain is of great interest to the field of regenerative medicine, which aims to develop therapies that use NSCs to replace lost neurons in the diseased or injured brain ^62^.

So far, the embryonic origin of adult RGLs in the DG has not been identified. It remains unknown whether adult RGL precursors contribute to neurogenesis during the development of the DG and then continue to be active into adulthood or whether a subset of NSCs are set aside during development, as they are in the SVZ, to then become reactivated during adulthood ( Figure 4A, B). To determine which model is correct, the fate choices of individual NSCs should be determined at different stages of development. It will also be important to examine whether the NSCs that give rise to the DG also generate neurons in other areas of the hippocampus or cortex or whether they become lineage-restricted at an early stage of development.

## Using single-cell analyses to investigate adult radial glia-like cells

Owing to the sparsity, heterogeneity, and dynamic nature of adult NSCs, it is difficult to study these cells using conventional population-level analyses. In order to identify different subtypes of RGLs in the adult DG, one has to examine the cells in the SGZ on a single-cell level. The last decade has seen a vast increase in new single-cell technologies, such as single-cell RNA sequencing, clonal lineage tracing, and *in vivo* imaging. Here, we discuss these techniques and how they might be used for the study of NSCs in the developing and adult DG at the single-cell level.

### Single-cell sequencing of transcriptomes and epigenomes

Recent technical advancements in single-cell transcriptome and epigenome profiling technologies have made it possible for researchers to commence deciphering heterogeneous populations of stem cells in different tissues, including NSCs ^63^. In both the embryonic and the adult brain, molecular signatures identified through single-cell RNA sequencing have been used to detect previously unknown cell types and to identify novel markers for subpopulations of NSCs.

In the developing human brain, the outer radial glia represent a population of cells which are thought to give rise to most cortical neurons. Though clearly important for the development of the human brain, the molecular features of these cells were not known. To address this question, researchers performed RNA sequencing, which has revealed a multitude of new markers for the outer radial glia ^64, 65^. The new markers have been used to identify outer radial glial cells in *in vitro* culture experiments, demonstrating the predictive accuracy of the data generated ^66^. In the adult DG, single-cell RNA sequencing of Nestin-CFP-expressing cells in the DG ^67^ revealed that, on the basis of their transcriptome, quiescent RGLs can be divided into different groups, which represent progressive stages in a developmental trajectory. Additionally, this study revealed the molecular signatures of the active RGLs and early IPCs. Markers which are strongly expressed in distinct groups of cells at specific time points, and no other cell types in the DG, will be good candidates for lineage-tracing experiments to determine the long-term behavior of these cells (see below).

The field of single-cell RNA sequencing is rapidly progressing. In these first studies, the number of sequenced cells numbered in the hundreds. But the development of new techniques, such as Drop-seq, means that many more cells can be sequenced at a reasonable cost ^68, 69^. Some populations of stem cells might be quite rare such that increasing the number of sequenced cells will increase the resolution and potentially lead to the discovery of new subpopulations. This, together with future improvements in sequencing depth and coverage, will further illuminate the complex heterogeneity of different stem cell populations.

In addition to RNA sequencing, which examines differences in transcriptomes, analysis of the epigenetic landscape of cells can further reveal differences between cell populations. Technologies such as bisulfite sequencing to determine DNA methylation ^70^; assay for transposase-accessible chromatin sequencing (ATAC-seq), which reveals chromatin accessibility ^71^; and analysis of chromosome structure on a single-cell level ^72^ are available to examine epigenetic regulation on a single-cell level.

Single-cell sequencing techniques are still in their infancy but are rapidly becoming more efficient and reliable. In the coming years, we might even be able to perform both RNA sequencing and multiple epigenome profilings on the same cell. In addition, there are recent developments of technologies for profiling epitranscriptomes and appreciation of their critical role in neurogenesis ^73^. These methodologies ultimately will reveal further layers of heterogeneity within NSC populations.

### Single-cell lineage tracing

While single-cell RNA sequencing may reveal novel markers for subpopulations of RGLs in the DG, it can reveal only the molecular signature of a transient state. Long-term lineage tracing is needed to determine the lineage potential of these subpopulations over time. Lineage tracing on a clonal level has been performed in the adult DG using the Nestin-CreER ^T2^ mouse line and has revealed that these RGLs can self-renew and generate both neurons and astrocytes ^13^. This technique has also been combined with genetic manipulations to examine the role of genes, such as *PTEN*, *sFRP3*, γ _2_-subunit-containing GABA _A_ receptors, and *NF1*, in regulating quiescent NSC behavior ^13, 15, 37, 74^.

Single-cell lineage tracing could also be used to characterize the behavior of different populations of stem cells in a tissue by using different CreER mouse lines. For example, although both Nestin and GLAST are expressed by most, if not all, RGLs at the protein level, the Nestin-CreER ^T2^ and GLAST-CreER ^T2^ mouse lines label RGLs with different behaviors at the population level ^28^. It would be interesting to compare these drivers in a clonal analysis experiment to investigate potential differences in fate choice or maintenance with higher resolution.

Single-cell lineage tracing is also a powerful tool for the study of brain development. Clonal analysis using the Mosaic Analysis with Double Markers (MADM) system has been used to examine the development of the cortex and thalamus ^75, 76^. The MADM system is a two-color system, in which cells in G _2_-X phase that express Cre recombinase undergo Cre-mediated inter-chromosomal recombination, which can lead to RFP expression in one daughter cell and GFP expression in the other daughter cell ^77^. These two sister cells and their progeny then can be traced separately over time to assess their fate choices. The MADM system would be very useful to determine the origin and behavior of NSCs during DG development. Cre-driver mouse lines need to be screened for labeling the developmental precursors of the RGLs.

### *In vivo* imaging

To get a complete understanding of stem cell behavior, researchers are now aiming to image stem cells *in vivo*. This would enable the examination of individual stem and progenitor cells over time in a living animal ^78^. Many technical hurdles remain before this can be done, especially when it comes to the DG, which is situated deep in the hippocampus.

Recent technical advancements for *in vivo* imaging have been performed in zebrafish, a teleost fish in which neurons are generated in many areas of the adult central nervous system ^79^. The brain of the teleost fish develops through outward bending or eversion with the result that the adult NSCs, which have radial glia-like morphology, have their soma on the outside of the brain, close to the surface, making the NSCs easier to visualize. Additionally, some zebrafish lines lack pigment, making them more transparent and thus enabling deep tissue imaging with high resolution, making it possible to image single NSCs over time in a live animal by using confocal microscopy. This technique has made it possible for researchers to study the fate choices made by individual NSCs in different brain areas for up to one month ^80, 81^.

Since the DG and SVZ are situated deep inside the mammalian brain, this makes them difficult to access for imaging without injuring the brain. Nevertheless, *in vivo* imaging has been used to study the behavior of adult-born neurons in the DG ^82^. In this study, investigators used the Nestin-CreER ^T2^ line crossed with a tdTomato reporter line and waited 6 weeks after tamoxifen injection, meaning that the tdTomato ^+^ cells were 6 weeks or younger. Calcium imaging was performed on these cells, and investigators found that newborn neurons actively participate in encoding information and are more active and less spatially tuned compared with the more mature granule cells. Another study used retrovirus to label and birth-date newborn neurons to examine dendritogenesis over time ^83^. Efforts should be made to image the behavior of NSCs in the adult DG, but this will have many technical challenges which need to be overcome. Possibly, the biggest hurdle will be that the DG is situated deep in the brain, under the cortex, and using current imaging strategies will undoubtedly lead to significant injury that might alter the behavior of the NSCs. Once these issues have been overcome, *in vivo* imaging will be a powerful tool to determine the behavior and characteristics of individual RGLs.

## Concluding remarks

Adult stem cell heterogeneity has garnered increasing attention in the last decade ^18, 84– 86^. Recent studies have shown that the NSCs in the SGZ of the DG can be distinguished by differences in their morphology, lineage potential, and function during tissue maintenance and repair. Future studies will be needed to determine whether these variations are due to the presence of multiple, restricted populations or to different states within the same population *in vivo*. Another important question is whether stem cell heterogeneity is established during development or adulthood, which will require a better understanding of the embryonic origin of the adult RGLs. Investigators should continue to use single-cell techniques as discussed in this review, such as clonal analysis and single-cell sequencing, to address these questions and to determine when and how stem cell heterogeneity is established in the adult DG.
